# Green Paper on Bio–Preparedness–general comments–

**Published:** 2010-11-25

**Authors:** M Sirbu

**Affiliations:** Attorney at law, Bucharest Bar Association Romania

**Keywords:** biopreparedness

## Abstract

The Commission's Green Paper on Bio–preparedness represents an important signal that the European Commission is actively involved in, working on issues related to bio–preparedness across all Member States and the international Community. In 2006, the Commission held two seminars on European Bio Preparedness and a workshop on Transport and Traceability of Bio materials. The results and recommendations emerging from these discussions have been inserted in this Green Paper. The document intends to stimulate a debate within and between the Member States and to launch a process of consultation on how to reduce biological risks and to enhance preparedness and response. All the national authorities responsible for risk prevention and response, human, animal and plant health, customs, civil protection, law enforcement authorities, the military, bio–industry, epidemiological and health communities, academic institutions and bioresearch institutes are therefore called to be involved, to contribute and to improve the ability of the EU to prevent, respond to and recover from a biological incident or deliberate criminal activity.

Weapons of mass destruction as WMD have gained an important place in recent years because of the destructive powers of such weapons and the devastating consequences upon population, which determine political and economic instability.

The nations must be prepared to prevent and to respond with the right capabilities to such WMD events.

We all know that even if the risk of bio–terrorist attack is statistically low, the consequences in case such an event occurs are catastrophic.

The biotechnology industry continues to expand globally and dual–use expertise and technology could become available to criminal political entities and terrorists. That's why the legal framework is meant to interdict and to limit to the minimum the possibility for the bioterrorists to acquire and gain lethal capabilities. Bio laboratories should be under control; there are untold number of such laboratories containing refined pathogen seed stocks.

If pathogens were diverted at the present in most places around the world, it is highly unlikely that law enforcers would be able to find out in time and to stop the catastrophe.  

**Weapons of Mass Destruction includes:**

- Highly hazardous chemical agents;
- Biological agents–microbes and toxins;
- Radiological agents capable of emitting alpha, beta and gamma radiation;
- Nuclear blasts

## Materials|methods

To provide a legal and correct image of what is really happening in this area of activity the present paper analyzes and comments on key principles of bio–preparedness and on issues that this document identified for the way forward, focusing on the definitions of the terms used in the Green Paper related to other EU documents.

## Discussions and results

Addressing the WMD issues means a global approach for a global response, taking into consideration the specificity of biological weapons. We need to focus on a holistic biological risk reduction approach and to combine therefore the 1972 Biological and Toxins Weapons Convention, the non proliferation suppliers group, Australia Group and public health assistance tools. This way we will have the possibility to link security with development for our own benefits.

Protecting the health and wellbeing of EU citizens is a top priority for the European Commission, and therefore it was adopted a Green Paper on bio –preparedness, inviting the major stake holders and all the national authorities of relevant parties to provide the Commission with input on how existing instruments can be improved to deal with biological threats and to be able to provide an effective prevention and response.

This document intends to stimulate a debate, to launch a process of consultation at European level on reducing the biological risks and to enhance preparedness and response capabilities.

The document launched a three month consultation period within public and private stake holders to respond to 35 topics mentioned in the document. These included prevention and protection measures required for the future, security of bio–labs, dissemination of pathogen research, the creative of professional codes of conduct and best practice guidelines surveillance capabilities, etc and pan –EU response and recovery mechanisms

The states are invited to work together to present concrete actions needed in order to improve the ability of EU to prevent, respond to and recover from a biological incident or deliberate criminal activity.

The feedback from the stakeholders is very important in order to evaluate the mechanisms and the legal frameworks which are already in force, how they work, to identify the gaps and to propose specific actions required in compliance with the provisions set out in article 5 of the EC Treaty. (to mention).

The aim of bio–preparedness is not to duplicate the legal framework set up regarding food and product safety, emergency measure in cases of accidents, but to complement this framework in order to improve security and the prevention of deliberate criminal acts, accidents as well as the response to naturally occurring outbreaks; to improve disease surveillance (an example is the network for epidemiological surveillance and control of communicable diseases in the Community setup by Decision  2119/98/EC of the European Parliament  and of the Council.), detection systems; to enhance the cross–border cooperation and communication; to facilitate international laboratory cooperation and to develop mechanisms for international sharing of medical countermeasures. 

Cross–border cooperation is essential for any effective preparedness strategy and response. Therefore the efforts of the states must be coordinated  in order to be able to reduce the biological risks.

Certain specific measures exist already in EU to ensure bio–safety and civil protection. These measures need to be adapted to cope with deliberate attacks.

There are regulations regarding the civil protection–Community Mechanism for civil protection assistance–Council Decision 2001/792/EC EURATOM, the Civil Protection Financial Instrument  was established in 2007. These documents underline the idea of a legal and financial framework for the reinforcement of the current activities. 

We have regulations to minimize the risks related to the contaminants in foodstuffs; there are legal instruments in the field of food safety and traceability, protection of workers from risks related to exposure to biological agents at work, enhancement of security, etc.

What are the key–principles of bio–preparedness?

- We need peer evaluation, awareness raising campaigns and supportive financial programs.
- The activities should build on existing structures and expects
- Private sector and research institutes should be involved through a Public–Private Security Dialogue. ( within ESRIF–EUROPEAN SECURITY RESEARCH AND INNOVATION FORUM )
- Members states authorities at national level should develop and implement a consistent approach within their jurisdictions which will benefit the EU as a whole.

Issues  the Green Paper identifies for the way forward

- Awareness about the existing legislative framework;
- Prevention and protection;
- Practical implementation  of safety standards and procedures;
- Existence and application of minimal security standards related to biological research;
- Professional code of conduct;
- Improving surveillance capacity;
- Response and recovery–cooperation between civilian health, civil protection and law enforcement authorities;
- Preserving and developing an European response to biological risks and threats.

How  we  define the terms used in the Green Paper?

**‘Preparedness’** = covers all aspects such as prevention, protection, prosecution of criminals/terrorists, surveillance, response and recovery; also the steps taken to minimize the threat of deliberate contamination of the food supply through biological agents and to protect against biological warfare which is defined as the deliberate use of micro–organisms or toxins to induce death or disease in humans animals or plants.

**‘Bio–security’** and **‘bio–safety’**  should be understood in a different way, according to the WHO Laboratory Bio–security guidance.

**‘Food safety’**= focuses in setting standards regarding the safety of food, good manufacturing practice and quality control of agricultural products at all steps of the processing chain.

**‘Food security’ **= is defined by WHO as access to sufficient, safe and nutritious food.

**‘Bio–Preparedness’** refers to strategies on how to reduce biological risks and to enhance preparedness and response to such risks. The aim of Bio–Preparedness is to improve the ability to prevent, respond to and recover from a biological incident or deliberate criminal activity.

The Commission adopted on 4 of August 2008 its staff working document **Synthesis of the replies to the green paper on bio–preparedness.**

A total of 82 replies were received. The results of the public consultation on bio–preparedness point to the conclusion that there is a consensus between the EU Member States to raise and tackle the issue of bio–preparedness at EU level. ( 23 – EU countries, 4 third countries, 4 representatives of regional authorities, 3 from regional organizations, 1 from office of the High Representatives personal for non–proliferation, 28 from private sector divided between associations, consortia and private companies, 14 from researchers and academics.

## Conclusions of the responses received

- the debate on bio–preparedness was welcome;
- it was considered important to develop concrete actions in this field;
- the idea of a more effective cross–sector cooperation was emphasized;
- the need of not duplicating the existing rules, guidelines, principles and standards;
- the need for an analyze of the existing gaps to start point additional initiatives;
- the private sector needs to get involved more in the field of bio–preparedness.

In parallel, the Commission set up a CBRN Task Force which brings together Member States and stakeholders to discuss the various questions raised in the Green Paper.

The **Bio Subgroup: Detection and Diagnosis (human, animal and plants)** aims to identify the concrete actions need to be taken in order to improve detection and diagnosis of biological substances threatening  humans, animals and plants. So, a concrete policy package could be put forward by the Commission in 2009.

On the basis of the Task Force meetings the Commission has started to draft an action bio–preparedness plan and will also draft another one on radiological and nuclear risk reduction.

Concepts which could be used for improvement of bio–detection:

- identifying work priorities on the detection field;
- developing minimum detection standards;
- establishing certification, testing and trialing schemes in the EU for biological detection;
- laboratory issues–networks, detection, identification;
- improving the exchange of information;
- making better use of detection technologies in specific locations;
- implementation
